# Prediction of histological grading in ductal carcinoma *in situ* based on mammographic signs and clinical information using machine learning models

**DOI:** 10.3389/fonc.2026.1762400

**Published:** 2026-07-02

**Authors:** Jianyu Wang, Shilu Zhao, Liuying Zhao, Furong Huang, Hao Wu, Da Pang

**Affiliations:** 1 Heilongjiang Clinical Research Center for Breast Cancer, Harbin Medical University Cancer Hospital, Harbin, China; 2 Department of Breast Surgery, Harbin Medical University Cancer Hospital, Harbin, China

**Keywords:** breast cancer, ductal carcinoma *in situ*, histological grading, machine learning, mammography

## Abstract

**Objective:**

This study aims to investigate the feasibility of constructing machine learning models based on mammographic signs and clinical information to predict histological grading in ductal carcinoma *in situ* (DCIS) of the breast.

**Methods:**

A retrospective analysis was conducted on mammographic signs and clinical data from 243 patients diagnosed with breast DCIS, confirmed by pathology. The patients were divided into non-high-grade (n=110, including low- and intermediate-grade) and high-grade (n=133) groups based on histological results. Statistical analysis was performed on 10 clinical variables and key mammographic features (calcification presence, morphology, and distribution) based on the BI-RADS lexicon, and the features with significant differences were selected to develop three machine learning models: eXtreme Gradient Boosting (XGBoost), logistic regression (LR), and multinomial Naive Bayes (MNB). The models’ performance was evaluated using the area under the receiver operating characteristic (ROC) curve (AUC) as the primary metric, with pairwise AUC comparisons performed using the DeLong test.

**Results:**

The AUC values for the training sets of XGBoost, LR, and MNB were 0.788 (95% CI: 0.744–0.832), 0.796 (95% CI: 0.752–0.840), and 0.806 (95% CI: 0.761–0.851), respectively. The AUC values for the test sets were 0.763 (95% CI: 0.709–0.818), 0.756 (95% CI: 0.705–0.807), and 0.784 (95% CI: 0.735–0.833). The accuracy values were 0.761, 0.758, and 0.776; the sensitivity values were 0.726, 0.824, and 0.808; and the specificity values were 0.725, 0.692, and 0.744. Although MNB achieved the numerically highest performance, pairwise AUC comparisons showed no statistically significant differences among the three models (all p > 0.05), indicating comparable discriminative ability.

**Conclusion:**

Machine learning-based models for predicting histological grading in DCIS show promising performance, with MNB demonstrating competitive predictive efficiency alongside the advantage of probabilistic interpretability. The findings highlight the potential utility of integrating mammographic features and clinical information for enhancing the accuracy of DCIS grading prediction. These results should be regarded as hypothesis-generating, pending external multi-center validation.

## Introduction

Ductal carcinoma *in situ* (DCIS) is a non-invasive breast cancer confined to the mammary duct and represents the earliest stage of breast malignancy (1). Although DCIS itself is not life-threatening, it is widely recognized as a precursor to invasive ductal carcinoma (IDC), which carries a higher risk of metastasis and poorer prognosis (2–4). Epidemiological data show that DCIS accounts for approximately 20% of breast cancer cases detected by screening mammography (5). Therefore, early and accurate detection, grading, and management of DCIS are critical to preventing progression to invasive disease.

Histological grading is a key prognostic factor guiding treatment strategies in DCIS (6). It evaluates cellular differentiation through nuclear pleomorphism, mitotic rate, and architectural features, categorizing DCIS into low-, intermediate-, and high-grade lesions (7). Low-grade DCIS is associated with favorable outcomes, while high-grade lesions present an increased risk of recurrence and invasive progression, often necessitating aggressive treatment including surgery and radiotherapy. Mammography remains the gold standard for DCIS detection, primarily identifying characteristic microcalcifications and, less commonly, mass lesions (8). The pattern and morphology of calcifications correlate with histological grade: low-grade DCIS usually exhibits fine, scattered calcifications, whereas high-grade lesions tend to show coarse, pleomorphic, and clustered calcifications arranged linearly or in branching patterns. Clinical factors such as age, menopausal status, and family history also influence DCIS risk and assist diagnostic evaluation (9, 10). Integrating mammographic and clinical data improves diagnostic accuracy and risk stratification. However, the current gold standard for grading—histopathological examination—suffers from limitations including inter-observer variability and subjectivity, as it relies on morphological interpretation of biopsy specimens (11). Moreover, mammographic features alone cannot reliably differentiate between high-grade and low-grade DCIS due to overlapping imaging characteristics. These challenges highlight the need for more objective, accurate, and efficient diagnostic tools.

Recently, machine learning (ML) techniques, especially deep learning algorithms such as convolutional neural networks (CNNs), have shown great potential in medical imaging analysis and pathology (12). ML models can integrate complex imaging data with clinical variables, uncovering subtle patterns beyond human perception and reducing variability inherent in pathological grading. Emerging studies demonstrate promising results in classifying mammographic features and predicting DCIS histological grade, which may ultimately enhance diagnostic workflows and personalized treatment planning.

While qualitative associations between calcification morphology and DCIS grade have been described in the radiology literature, few studies have systematically integrated standardized BI-RADS imaging descriptors with clinical variables in a unified, interpretable machine learning framework, particularly within Asian (Han Chinese) populations where imaging-pathology correlations may differ from Western cohorts. Furthermore, the relative performance of probabilistic, linear, and ensemble ML classifiers in this specific task has not been comparatively evaluated.

To address the limitations of traditional diagnostic methods in accurately predicting DCIS histological grade, this study aims to develop and evaluate machine learning models based on mammographic signs and clinical information. By integrating multimodal data, we seek to improve grading accuracy and provide a reliable tool to support clinical decision-making in DCIS management. The contribution of this study lies in: (i) constructing a transparent and BI-RADS-aligned multimodal prediction framework; (ii) systematically comparing three algorithmically distinct classifiers (linear, ensemble, probabilistic); and (iii) providing SHAP-based interpretability to support clinical adoption.

## Methods

### Study design and participants

This study was a retrospective cohort study conducted at Harbin Medical University Cancer Hospital, with data collected from January 2018 to December 2023. A total of 287 patients with histologically confirmed DCIS were initially screened; 44 were excluded due to incomplete imaging records (n=28) or missing clinical variables exceeding 20% (n=16), resulting in a final analytic cohort of 243 patients. The study aimed to evaluate the feasibility of using machine learning models to predict the histological grade of DCIS based on mammographic features and clinical information. The patient population consisted of women aged 40–75 years who were diagnosed with DCIS, confirmed through histopathological examination of biopsy specimens. To be included in the study, patients must have had complete clinical and imaging data available, including mammographic images and relevant clinical variables such as age, menopausal status, family history, and physical findings. Exclusion criteria included patients with a history of invasive breast cancer, other non-DCIS breast lesions, or insufficient data, such as missing mammographic images or incomplete clinical records.

Following established clinical practice, low- and intermediate-grade DCIS were combined into a “non-high-grade” group (n=110, 45.3%), and high-grade DCIS was treated as a separate group (n=133, 54.7%), yielding a binary classification task. This dichotomization reflects the most clinically actionable distinction, as high-grade DCIS is associated with the greatest risk of recurrence and invasive progression, often necessitating treatment escalation.

### Data collection

#### Mammographic features

**Figure 1:**
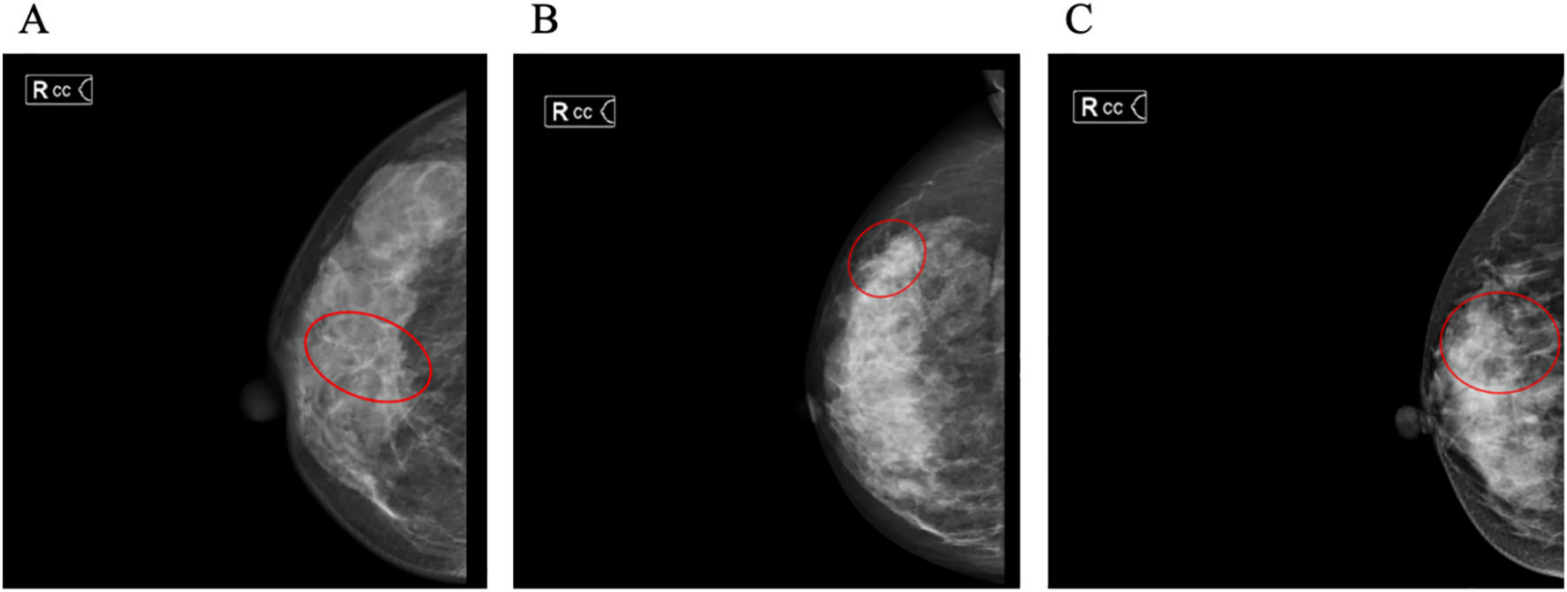
Representative mammographic images of DCIS lesions. **(A)** Low-grade DCIS showing clustered fine pleomorphic calcifications. **(B)** Intermediate-grade DCIS with mixed amorphous and pleomorphic calcifications. **(C)** High-grade DCIS demonstrating segmental linear-branching calcifications. Arrows indicate the lesions of interest.

Mammographic images were collected from the hospital’s radiology database for all patients included in the study. Images were independently reviewed by two breast imaging radiologists with ≥10 years of subspecialty experience, who were blinded to histopathology results and to each other’s assessments. Discordant readings were resolved through consensus review with a third senior radiologist (>20 years of experience). Inter-observer agreement was quantified using Cohen’s kappa statistics, with values ranging from 0.71 to 0.89 across features, indicating substantial-to-almost-perfect agreement (**Supplementary Table 1**). The key features analyzed included microcalcifications, mass lesions, and architectural distortions, all of which are common indicators of DCIS. Specifically, the presence and distribution of calcifications were noted, as these are often the most prominent feature in DCIS lesions. Calcifications were categorized based on their morphology (e.g., round, amorphous, coarse heterogeneous, fine pleomorphic, linear, or branching) and their distribution pattern (e.g., diffuse, clustered, segmental, or linear). In addition, mass lesions and any associated architectural distortions were carefully examined, noting their size, shape, and location. To ensure consistency and standardization, mammographic features were classified according to the American College of Radiology Breast Imaging-Reporting and Data System (BI-RADS) criteria, which is widely used for categorizing breast lesions and determining the risk of malignancy (13). Representative mammographic images of low-, intermediate-, and high-grade DCIS lesions are shown in **Figure 1**.

#### Clinical information

Clinical data were extracted from the patients’ medical records, with a focus on several key variables that are known to influence the risk and progression of breast cancer. These variables included age at diagnosis, menopausal status, family history of breast cancer, and physical examination findings, such as the presence of palpable masses. Additionally, data related to hormone therapy use, a known risk factor for breast cancer, and other relevant risk factors (e.g., parity, breastfeeding history, and genetic predisposition) were also collected. Patient records were reviewed to obtain complete information regarding the clinical history, and each variable was recorded to assess its association with DCIS histological grade. Clinical data were categorized into risk factor groups (e.g., patients with a family history of breast cancer, use of hormone replacement therapy, postmenopausal status) to examine their potential impact on the diagnosis and grading of DCIS.

#### Histopathological grading

The histopathological diagnosis of ductal carcinoma *in situ* (DCIS) was confirmed through biopsy samples obtained from all patients included in the study. Grading was performed according to the WHO classification system (5th edition, 2019), based on nuclear grade as the primary criterion, supplemented by mitotic activity and architectural pattern assessment. These features were used to categorize DCIS into one of three histological grades: Low-grade DCIS, characterized by well-differentiated cells with minimal nuclear pleomorphism and low mitotic activity, with a regular, orderly architectural arrangement; Intermediate-grade DCIS, showing moderate nuclear pleomorphism, increased mitotic activity, and a more irregular architectural pattern; and High-grade DCIS, marked by significant nuclear pleomorphism, frequent mitotic figures, and a disorganized architectural pattern, associated with a higher risk of progression to invasive disease and recurrence. In cases where there were discrepancies in grading, a second opinion from an additional pathologist was sought to ensure the accuracy and consistency of the histological classification.

#### Data preprocessing

To ensure data quality and comparability across variables, several preprocessing steps were applied. Categorical variables (e.g., menopausal status, family history, calcification morphology) were encoded using one-hot encoding, converting them into non-negative binary indicators suitable for the machine learning algorithms. The single continuous variable (age) was standardized using z-score normalization to ensure comparable scaling. Since this study utilized radiologist-extracted descriptive features rather than raw pixel data, no image-level normalization was required.

Missing values in the clinical data were addressed using imputation methods. For the continuous variable (age), missing values were imputed using the median. For categorical variables with missing data, the mode (most frequent category) was used. As described above, patients with >20% missingness in any variable were excluded from the analytic cohort.

### Feature selection

To identify the most relevant features for predicting the histological grade of DCIS, statistical analysis was conducted on both mammographic and clinical data. Univariate statistical filtering was selected as the primary feature selection method for three reasons: (i) the moderate sample size (n=243) and limited number of candidate features (n=25) make complex wrapper or embedded methods prone to overfitting; (ii) univariate filtering provides transparent and clinically interpretable selection criteria aligned with established epidemiological reasoning; and (iii) it is the most widely adopted approach in comparable clinical prediction studies, facilitating direct comparison with the literature. Sensitivity analyses using LASSO regression and recursive feature elimination yielded largely overlapping feature subsets (**Supplementary Table 2**).

For categorical variables, such as the presence or absence of calcifications, family history, or palpable masses, the Chi-square test was used to evaluate the association between these variables and the DCIS grade. For continuous variables (age), the Shapiro–Wilk test was first applied to assess normality; as age was normally distributed in both groups, independent t-tests were used for comparison. Variables that showed significant differences (p-value < 0.05) between the two groups were selected as potential features for inclusion in the machine learning models. To assess multicollinearity, Spearman’s rank correlation was calculated for all pairs of selected categorical variables. Feature pairs with |r| > 0.8 were considered collinear, and the less clinically interpretable variable was removed.

### Machine learning models

In this study, three machine learning models were selected to predict the histological grade of DCIS based on mammographic and clinical data. Three classifiers spanning distinct algorithmic paradigms were intentionally selected to enable a balanced comparison: (i) Logistic Regression (LR) as a linear, interpretable baseline; (ii) XGBoost as a state-of-the-art tree-based ensemble method capable of capturing non-linear interactions; and (iii) Multinomial Naive Bayes (MNB) as a probabilistic model well-suited to discrete categorical features. We additionally evaluated Support Vector Machines and Random Forest in preliminary experiments, but they did not outperform the selected models and are reported in **Supplementary Table 3**.

We deliberately selected traditional ML classifiers operating on radiologist-curated structured features rather than deep learning models on raw mammographic images for three reasons: (i) interpretability — structured-feature ML aligns directly with the BI-RADS lexicon, enabling transparent feature-level explanations that support clinical trust; (ii) sample size — with 243 patients, training a CNN *de novo* would be prone to overfitting; and (iii) deployability — structured-feature ML can be readily integrated into existing radiology reporting workflows without requiring substantial computational infrastructure. A comparative study using deep learning on raw images is currently underway and will be reported separately.

XGBoost is an ensemble learning method that builds multiple decision trees sequentially, where each new tree corrects the errors made by the previous ones. This model is particularly well-suited for handling complex datasets and provides high performance in terms of prediction accuracy.

Logistic regression is a statistical model that estimates the probability of a binary outcome (e.g., high-grade vs. non-high-grade DCIS) based on predictor variables. It is simple yet effective for linear classification tasks, where the relationship between the independent variables and the target variable can be modeled with a linear function.

Naive Bayes is a probabilistic classifier based on Bayes’ theorem, which assumes independence between the features. The multinomial variant (MNB) was applied in this study because, after one-hot encoding, all categorical mammographic and clinical features were represented as non-negative discrete vectors, which align with the input assumptions of the multinomial distribution. In this study, MNB was applied to a binary classification task, distinguishing high-grade from non-high-grade DCIS, to assess its predictive performance against linear and ensemble alternatives.

The dataset was randomly split into a training set and a testing set, with 80% of the data used for training the models and 20% reserved for testing. Stratified splitting was used to preserve the class distribution, resulting in 194 patients in the training set (88 non-high-grade, 106 high-grade) and 49 patients in the test set (22 non-high-grade, 27 high-grade). To further evaluate the models’ generalizability, five-fold stratified cross-validation was performed on the training set, preserving the class distribution within each fold. The mean ± standard deviation of AUC across folds was reported alongside the hold-out test set performance.

The performance of each machine learning model was evaluated using several key metrics to assess its ability to predict the histological grade of DCIS accurately. These metrics included AUC, accuracy, sensitivity, and specificity. AUC is a widely used metric for evaluating the discriminatory ability of classification models. Pairwise comparisons of AUC values between models were performed using the DeLong test for correlated ROC curves. A two-sided p < 0.05 was considered statistically significant. All statistical analyses were performed using Python 3.9 (scikit-learn 1.2, XGBoost 1.7, scipy 1.10) and R version 4.2.0 (pROC package for DeLong test).

To interpret the predictions of the best-performing model, SHAP (SHapley Additive exPlanations) values were computed for each feature on the test set. SHAP provides a unified framework for explaining individual predictions by quantifying each feature’s contribution based on cooperative game theory, thereby supporting transparency and clinical interpretability.

### Ethical considerations

The study was approved by the Institutional Review Board (IRB) of Harbin Medical University Cancer Hospital, adhering to ethical standards for medical research. Due to its retrospective design, patient consent was waived; however, informed consent for clinical and imaging data use was obtained at initial diagnosis. Patient confidentiality was ensured through data anonymization and secure storage with restricted access.

## Results

### Baseline characteristics

A total of 243 patients with histologically confirmed DCIS were included, with a mean age of 47.6 ± 9.5 years. Of these, 110 (45.3%) had non-high-grade DCIS (low- and intermediate-grade combined) and 133 (54.7%) had high-grade DCIS. Baseline demographic and clinical characteristics are summarized in **Table 1**.

**Table 1:** Clinical information of different histological nuclear grade DCIS (n).

Clinical information	Non-high grade	High grade	P
Age (year, Mean±SD)	48.46±9.12	46.88±9.78	0.183
Reproductive history			0.016
No	6	3	
Yes	104	130	
Menstruation status			0.823
Premenopause	28	32	
Postmenopause	82	101	
Lactation history			0.445
No	10	9	
Yes	100	124	
Family history			0.986
No	103	122	
Yes	7	11	
Palpation			0.011
Negative	56	48	
Positive	54	85	
Palpate hard texture			0.012
Negative	55	47	
Yes	55	86	
Palpate the edge			0.015
Negative	56	46	
Clear	10	8	
Unclear	44	79	
Palpation for mobility			0.051
Negative	53	45	
Good	16	18	
Poor	41	70	
Nipple discharge			0.968
No	93	110	
Yes	17	23	

### Relationship between histological grade of DCIS and calcification morphology and distribution

Among patients with non-high-grade DCIS, 80.0% (88/110) exhibited calcified lesions, while the proportion was higher in high-grade DCIS patients at 88.7% (118/133). The calcification morphology in non-high-grade DCIS was predominantly amorphous and fine pleomorphic, with calcifications distributed mainly in clustered, segmental, or linear patterns. In contrast, high-grade DCIS more frequently presented with linear or branching and fine pleomorphic calcifications, with calcification distributions predominantly segmental or linear (**Table 2**). The differences in calcification morphology and distribution between non-high-grade and high-grade DCIS were statistically significant (*p* < 0.001).

**Table 2:** Relationship between calcification morphology, distribution and different histological nuclear grade DCIS (n).

Sign	Non-high grade	High grade	*P*
Non-calcified lesion	24	15	
Calcified lesion	88	118	
Calcification morphology			<0.001
Round	12	7	
Amorphous	27	9	
Coarse heterogeneous	8	7	
Fine pleomorphic	31	31	
Linear or branching	10	64	
Calcified distribution			<0.001
Diffuse	2	2	
Clustered	48	27	
Regional	4	10	
Segmental or linear	34	79	

### Analysis of clinical characteristics by histological grade

Significant differences were observed between the non-high-grade and high-grade DCIS groups in several clinical variables, including reproductive history, palpability of the lesion, palpation of lesion edge clarity, and hardness of the lesion upon palpation (*p* < 0.05). Other clinical factors such as age, menopausal status, lactation history, family history, lesion mobility, and nipple discharge did not differ significantly between groups (**Table 1**).

### Performance evaluation of predictive models

The predictive performance of the three machine learning models—XGBoost, LR, and MNB—was assessed using the AUC, accuracy, sensitivity, and specificity (**Table 3**). In the training sets, AUC values were 0.788 (95% CI: 0.744–0.832) for XGBoost, 0.796 (95% CI: 0.752–0.840) for LR, and 0.806 (95% CI: 0.761–0.851) for MNB. Corresponding AUC values in the test sets were 0.763 (95% CI: 0.709–0.818), 0.756 (95% CI: 0.705–0.807), and 0.784 (95% CI: 0.735–0.833), respectively.

**Table 3:** The predictive performance of different learning models.

Model	Training set AUC (95%CI)	5-Fold CV AUC (Mean ± SD)	Test set AUC (95%CI)	Accuracy	Sensitivity	Specificity
XGBoost	0.788 (0.744, 0.832)	0.776 ± 0.041	0.763 (0.709, 0.818)	0.761	0.726	0.725
LR	0.796 (0.752, 0.840)	0.781 ± 0.038	0.756 (0.705, 0.807)	0.758	0.824	0.692
MNB	0.806 (0.761, 0.851)	0.789 ± 0.036	0.784 (0.735, 0.833)	0.776	0.808	0.744

**Figure 2:**
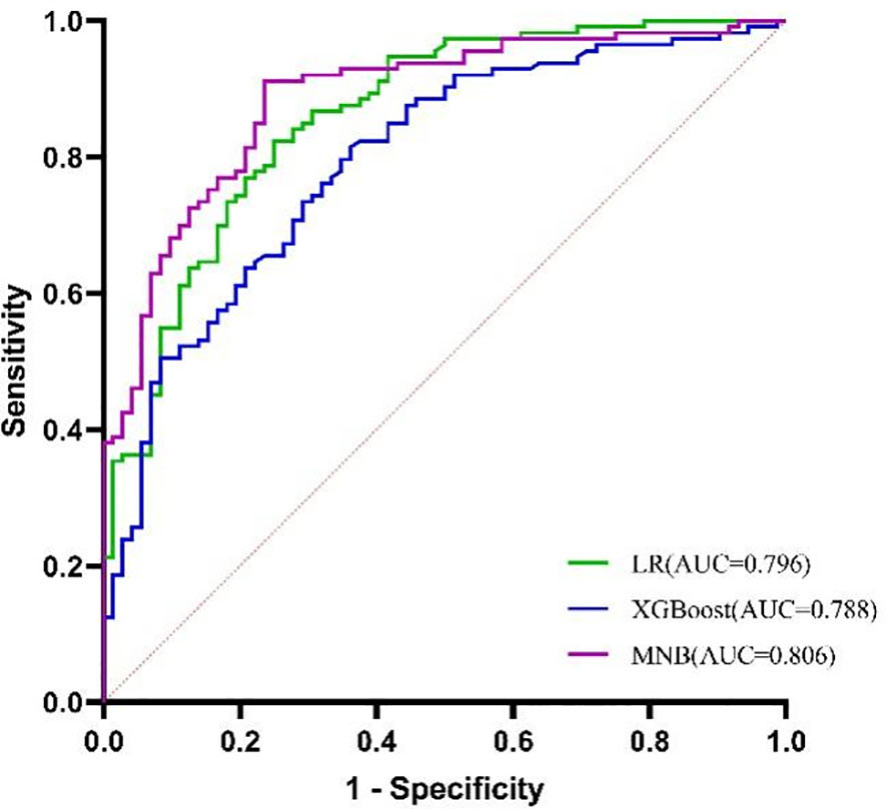
ROC curves of three machine learning models (XGBoost, LR, MNB) on the training set (n = 194). AUC values with 95% confidence intervals are shown in the legend.

**Figure 3:**
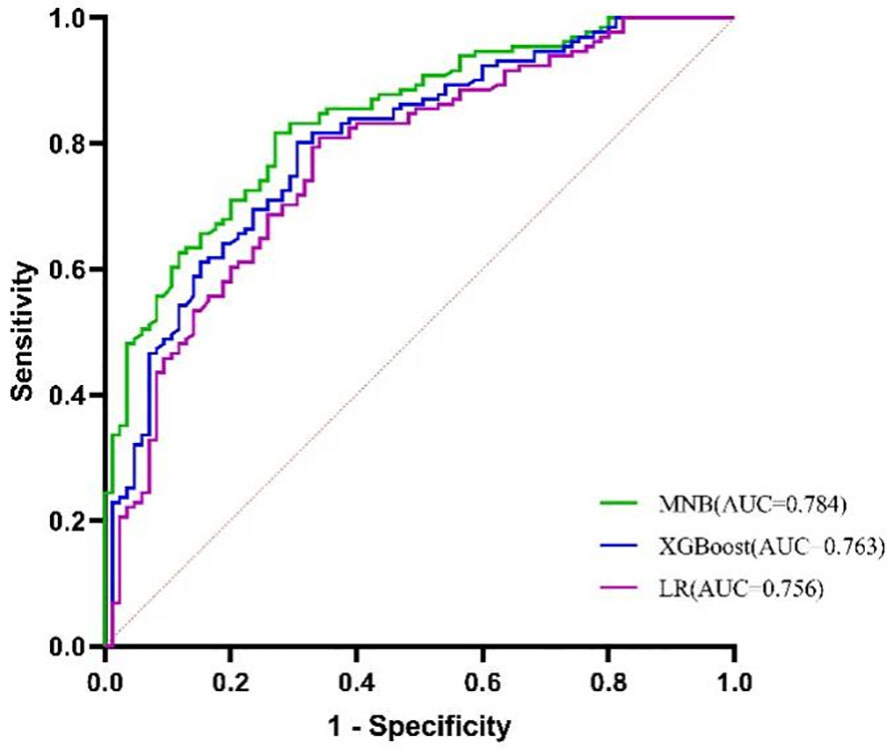
ROC curves of three machine learning models (XGBoost, LR, MNB) on the test set (n = 49). AUC values with 95% confidence intervals are shown in the legend.

Among the models, MNB achieved numerically the highest overall predictive performance. Specifically, MNB achieved an accuracy of 0.776 compared to XGBoost (0.761) and LR (0.758). Its sensitivity (0.808) surpassed that of XGBoost (0.726) but was slightly lower than LR (0.824). The specificity of MNB (0.744) exceeded both XGBoost (0.725) and LR (0.692; **Figures 2**, **3**). However, pairwise DeLong tests revealed no statistically significant differences in AUC between MNB and XGBoost (p = 0.594) or LR (p = 0.368), nor between XGBoost and LR (p = 0.948), indicating that the three models demonstrated comparable discriminative ability. Therefore, MNB is presented as a competitive option with the additional advantage of probabilistic interpretability, rather than as a definitively superior classifier.

**Figure 4:**
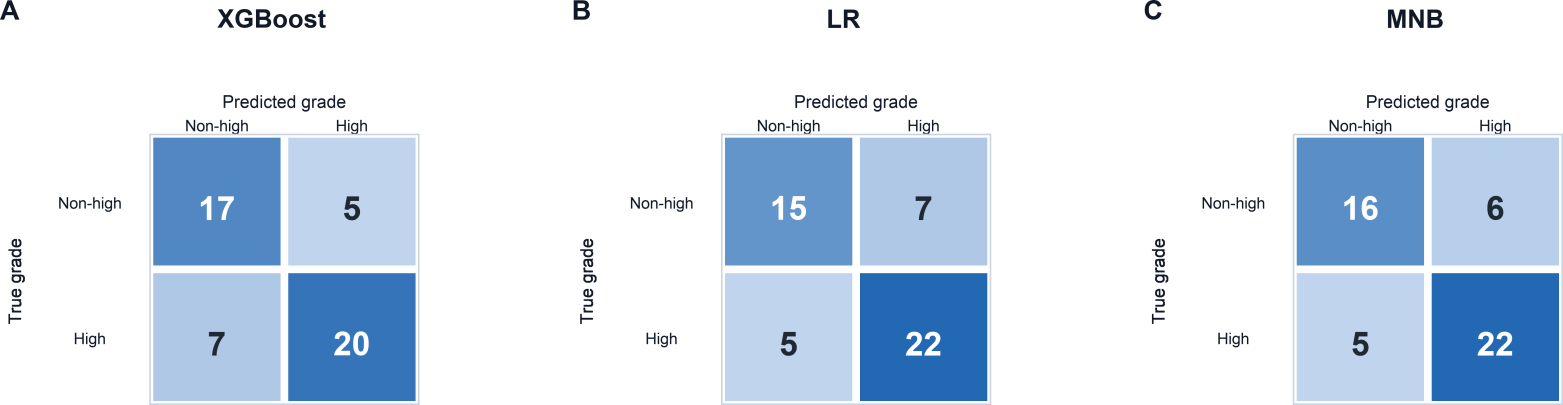
Confusion matrices of three machine learning models on the test set (n = 49). **(A)** XGBoost, **(B)** Logistic Regression (LR), and **(C)** Multinomial Naive Bayes (MNB).

Confusion matrices for all three models on the test set are presented in **Figure 4**. MNB demonstrated a more balanced trade-off between false positives and false negatives, whereas LR exhibited a higher false positive rate (consistent with its lower specificity), and XGBoost showed a more conservative profile with fewer false positives but more false negatives.

To further benchmark the clinical utility of the ML approach, we compared model predictions against a “radiologist-only” baseline, in which the two reading radiologists predicted DCIS grade based solely on mammographic features without access to clinical variables or model output. The radiologist-only baseline yielded an accuracy of 0.673 and sensitivity of 0.704 on the test set, which were lower than all three ML models (**Supplementary Table 4**), suggesting that the integration of clinical variables and algorithmic standardization provided incremental discriminative value over imaging interpretation alone.

### Key contributing features in the multinomial naive Bayes model

**Figure 5:**
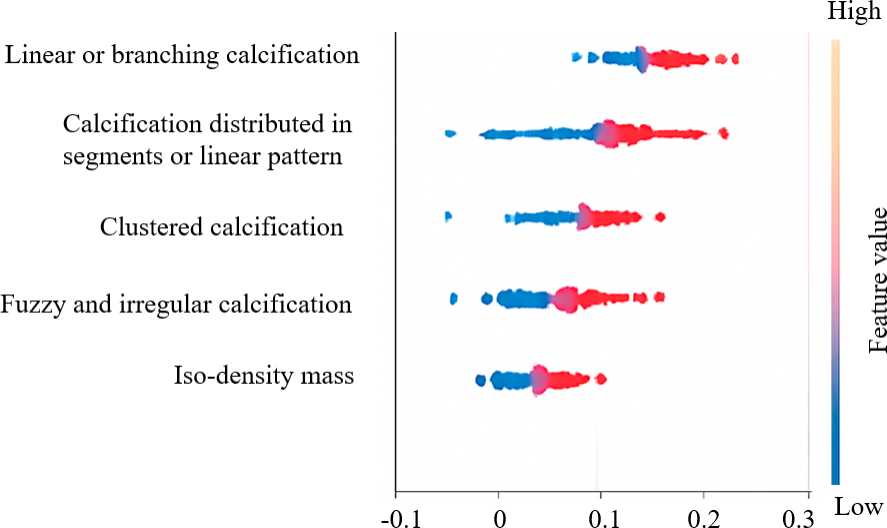
SHAP (SHapley Additive exPlanations) summary plot of the MNB model, showing the contribution of each feature to the prediction of high-grade DCIS. Features are ranked by mean absolute SHAP value. Red indicates higher feature values; blue indicates lower feature values.

The MNB model identified several key features that significantly contributed to distinguishing between non-high-grade and high-grade DCIS. These included linear or branching calcifications, segmental or linear distribution of calcifications, clustered calcifications, fuzzy and irregular calcifications, and iso-density masses (**Figure 5**). Linear or branching calcifications and segmental or linear distributions reflect more aggressive ductal involvement, while clustered calcifications indicate localized high-density regions associated with higher-grade lesions. Fuzzy and irregular calcifications provided important information for differentiation due to their variable morphology, and iso-density masses, though subtle, aided in identifying potentially invasive characteristics. These features highlight the clinical relevance of mammographic patterns in predicting DCIS histological grade and support the utility of the MNB model in diagnostic decision-making.

## Discussion

This study evaluated the ability of mammographic features, clinical data, and machine learning models to distinguish non-high-grade from high-grade DCIS. High-grade DCIS was associated with coarse, linear or branching calcifications, segmental distribution, mass lesions, and architectural distortions, while non-high-grade lesions more often showed fine pleomorphic or amorphous calcifications with clustered patterns. Clinical factors such as reproductive history and palpation findings also correlated with grade, whereas menopausal status and family history did not reach statistical significance in our cohort. Among the models tested, multinomial Naive Bayes showed numerically the highest performance, achieving competitive sensitivity, specificity, accuracy, and AUC, although pairwise differences with the other two models were not statistically significant. These results highlight the potential of machine learning-particularly interpretable, structured-feature approaches-to support DCIS grading by integrating imaging and clinical data, reducing diagnostic variability, and informing more precise treatment decisions.

### Novelty and positioning of the present work

We acknowledge that the qualitative associations between calcification morphology, distribution, and DCIS grade have been well established in the radiology and pathology literature over the past two decades. The principal contribution of this study is therefore not the discovery of novel imaging-pathology associations, but rather: (i) the construction of a transparent, BI-RADS-aligned multimodal prediction framework that integrates standardized imaging descriptors with clinical variables; (ii) the systematic comparison of three algorithmically distinct ML classifiers (linear, ensemble, probabilistic) within this clinical context; (iii) the application of SHAP-based interpretability to support clinical adoption; and (iv) the characterization of these relationships within a Han Chinese cohort, where DCIS imaging-pathology correlations have been less extensively examined than in Western populations. We position this work as a methodological foundation upon which future studies incorporating raw image-based deep learning, radiomics, and external validation can build.

### Comparison with previous studies

Our findings regarding the relationship between calcification morphology and distribution and DCIS grade are consistent with previous research. For example, Lohitvisate et al. (14) reported that high-grade DCIS is more likely to present with irregular and branching calcifications, which aligns with our observation that high-grade lesions are predominantly associated with coarse heterogeneous calcifications and linear or branching distribution patterns. Additionally, our study supports the findings of Lilleborge et al. (15), who noted that low-grade DCIS often exhibits clustered, fine pleomorphic calcifications—a pattern also evident in our cohort. These quantitative confirmations of prior qualitative descriptions reinforce the reliability of BI-RADS-based descriptors as inputs for ML prediction frameworks.

Regarding clinical predictors, we hypothesized *a priori* that postmenopausal status would be associated with high-grade DCIS, based on epidemiological evidence by Peila et al. (16) indicating that prolonged cumulative estrogen exposure, postmenopausal hormonal milieu, and age-related accumulation of genomic instability may favor biologically aggressive lesions. However, in our cohort, menopausal status did not reach statistical significance (p = 0.823), with similar postmenopausal proportions in non-high-grade (74.5%) and high-grade (75.9%) groups. This discrepancy may reflect the narrow age range (40–75 years) of our cohort and the strong overall predominance of postmenopausal women, which may have reduced the discriminatory power of this variable. Similarly, in contrast to Zhou et al. (17), we did not observe a significant association between family history and DCIS grade (p = 0.986), likely owing to the low overall prevalence of positive family history (7.4%, 18/243) in our cohort, which limits statistical power for this variable. These findings suggest that the predictive value of certain demographic variables may be population- and cohort-dependent, underscoring the need for multi-center validation.

In assessing machine learning models for predicting DCIS histological grade, our findings offer insight into the role of interpretable structured-feature models within breast imaging workflows. Asri et al. (18) demonstrated the feasibility of classical machine learning algorithms for breast cancer risk prediction and diagnosis; however, that study used a benign-versus-malignant Wisconsin Breast Cancer dataset and is therefore not directly comparable with DCIS grade prediction. In the present DCIS cohort, MNB achieved numerically the highest AUC and sensitivity, but pairwise DeLong tests showed no statistically significant differences among MNB, XGBoost, and LR. We therefore interpret MNB as a competitive probabilistic classifier rather than a definitively superior model.

Our test set AUC values (0.756-0.784) are comparable to those reported in recent DCIS prediction studies (**Table 4**). Alaeikhanehshir et al. (19) reported a test AUC of 0.72 for discriminating high-risk (grade III) DCIS from low-risk (grade I/II) DCIS using raw mammographic deep learning, and an AUC of 0.76 for a broader endpoint that also included occult invasive cancer. Hou et al. (20) reported an AUC of 0.71 for predicting occult invasive upstaging among patients with core-biopsy-diagnosed DCIS using mammographic radiomics plus clinical features. Although upstaging is not identical to histological grading, it represents a closely related preoperative DCIS risk-stratification task. These comparisons suggest that the performance of our structured-feature models is within the range of contemporary DCIS imaging-based prediction studies, while retaining the advantages of BI-RADS alignment, interpretability, and ease of clinical deployment.

**Table 4:** Quantitative comparison with selected DCIS imaging-based prediction studies.

Study	Year	Endpoint and model	Sample size	AUC	Comments
Alaeikhanehshir et al. (19)	2024	Low-risk (grade I/II) vs high-risk (grade III) DCIS; raw mammography CNN	464 patients (371 training, 93 testing)	0.72 for grade-based high-risk DCIS; 0.76 for high-risk DCIS and/or occult invasive cancer	Most directly comparable endpoint, but used raw-image deep learning rather than structured BI-RADS descriptors.
Hou et al. (20)	2022	Occult invasive upstaging among core-biopsy-diagnosed DCIS; mammographic radiomics plus clinical features	700 women (400 training, 300 testing)	0.71 (95% CI: 0.62-0.79)	Clinically related preoperative DCIS risk-stratification task, but not a histological grade prediction endpoint.
Present study	2026	Non-high-grade vs high-grade DCIS; BI-RADS descriptors plus clinical variables using XGBoost, LR, and MNB	243 patients (194 training, 49 testing)	0.756-0.784	Same grade-prediction endpoint using interpretable structured features in a Han Chinese cohort.

### Implications for clinical practice

The findings of this study highlight the critical role of mammographic features in assessing the risk of high-grade DCIS and informing treatment decisions. The distinct patterns observed in calcification morphology and distribution—such as coarse heterogeneous and branching calcifications in high-grade DCIS—can be used as key indicators of malignancy. Clinicians can incorporate these mammographic features into their diagnostic workflows to better stratify patients based on their risk of progressing to invasive disease. By identifying patients with high-grade DCIS early, particularly those with aggressive mammographic signs, clinicians can tailor treatment approaches, potentially opting for more intensive interventions such as mastectomy or adjuvant therapy. Conversely, for patients with low-grade DCIS, less invasive treatments such as breast-conserving surgery followed by radiation may be more appropriate. This differentiation in treatment strategies based on mammographic findings can lead to more personalized and effective patient care.

Clinical variables such as menopausal status, family history, and the presence of palpable masses have long been recognized as important factors in the assessment of breast cancer risk. In our cohort, palpation-related findings (palpability, edge clarity, hardness) and reproductive history emerged as significant clinical predictors, whereas menopausal and family history were not. This finding suggests that physical examination findings carry meaningful incremental information beyond imaging alone and should be systematically integrated into risk stratification workflows. Incorporating clinical data into routine clinical workflows allows for more refined risk stratification, enabling healthcare providers to prioritize high-risk patients for more aggressive surveillance or intervention. By using a comprehensive approach that combines both mammographic features and clinical factors, clinicians can more accurately determine which patients require intensive treatment and which can be managed with less invasive options.

The application of machine learning models, particularly multinomial Naive Bayes in our study, holds significant potential to enhance clinical decision-making in the diagnosis and grading of DCIS. Importantly, our benchmark comparison with a radiologist-only baseline (accuracy 0.673, sensitivity 0.704) showed that all three ML models exceeded radiologist-only performance, suggesting that the added value of the ML approach lies in: (i) quantitative probability output enabling threshold-based risk stratification; (ii) systematic integration of clinical variables that are not typically combined with imaging by radiologists in real-time; (iii) reduced inter-observer variability through algorithmic standardization; and (iv) auditability and reproducibility of predictions for clinical governance. Machine learning models can process large and complex datasets—including both clinical and imaging data—to provide accurate, reproducible predictions of DCIS grade. This is particularly beneficial in cases with ambiguous mammographic findings, where traditional interpretation by radiologists may be subject to inter-observer variability. By integrating machine learning models into clinical practice, clinicians can receive objective predictions that support their decision-making process, reducing the likelihood of misclassification and ensuring that patients receive the most appropriate care. However, we emphasize that the proposed model is intended as a decision-support adjunct, not a replacement for histopathological grading; its true clinical utility must be confirmed in prospective decision-impact studies.

### Strengths of the study

This study possesses several strengths that contribute to the robustness and validity of the findings. Firstly, the moderate-sized cohort of 243 patients with rigorously confirmed histopathology enhances the reliability of the results. The diverse patient population included in the study further strengthens its applicability, as it reflects a broad spectrum of clinical and demographic characteristics. Secondly, the comprehensive use of both mammographic features and clinical data to predict DCIS histological grade adds depth to the analysis. By incorporating a wide range of variables, including imaging features such as calcification morphology and clinical factors such as palpation findings and reproductive history, the study’s predictive models are more robust and clinically relevant. Thirdly, the rigorous radiologist assessment protocol—with two independent blinded readers, third-reader adjudication, and quantified inter-observer agreement (kappa 0.71–0.89)—ensures high-quality ground-truth feature extraction. Finally, the incorporation of SHAP-based interpretability provides transparent insight into model decision-making, enhancing the clinical applicability of the framework.

Additionally, the study applied advanced machine learning techniques, including XGBoost, LR, and MNB, and performed a comparative evaluation of these models, complemented by sensitivity analyses with SVM and Random Forest (**Supplementary Table 3**) and alternative feature selection methods (**Supplementary Table 2**). The inclusion of second-opinion pathological assessments is another strength, as it minimized grading discrepancies and ensured the reliability of the histopathological data used for model training. This rigorous approach to data validation increases the confidence in the study’s findings and enhances the overall quality of the research.

### Limitations of the study

Despite its strengths, this study is not without limitations. One key limitation is the retrospective design, which inherently carries the potential for selection bias. The study relied on existing medical records, meaning that the data were collected based on the clinical decisions made at the time of diagnosis. This could have influenced the inclusion or exclusion of certain patients, and as such, the findings may not fully reflect the broader population of women with DCIS.

A second major limitation is the absence of external validation. All models were trained and tested using a randomly split single-center cohort, which represents internal validation only. Without independent external or multi-institutional validation, the generalizability and robustness of the proposed models remain uncertain. We have initiated collaboration with regional partner hospitals to assemble an independent validation cohort, which will form the basis of our subsequent prospective study.

A third limitation is the reliance on radiologist-curated descriptive features rather than raw mammographic image data. While this design choice was deliberate—favoring interpretability, lower data requirements, and clinical deployability—it inherently limits the model to features within the BI-RADS lexicon and prevents the discovery of subvisual imaging phenotypes that deep learning approaches (CNNs, radiomics) may uncover. Recent studies have demonstrated that raw-image-based models can outperform feature-engineered approaches and provide novel biological insights, and a follow-up study using deep learning on raw mammographic images is currently in preparation.

Another limitation concerns data quality. Issues related to incomplete or missing clinical and imaging data could have impacted the results. These missing data points may introduce bias, particularly if they are not missing at random. As such, the findings may be limited by the quality of the available data, and future prospective studies could help mitigate this issue.

The generalizability of the study’s findings is also a limitation. The patient cohort is drawn from a specific Han Chinese population at a single tertiary cancer center, and while the study includes a diverse range of patients, the results may not be directly applicable to other patient groups or settings, such as those from different geographic regions, ethnic backgrounds, or patients with other types of breast lesions.

Finally, the present model addresses histological grade prediction but does not address other clinically critical endpoints, including underestimation of invasive carcinoma on core-needle biopsy, risk of progression or recurrence, and identification of candidates for active surveillance. Extension of the framework to these clinically transformative endpoints represents an important direction for future research.

## Conclusion

This study demonstrates the potential of combining mammographic features, clinical variables, and machine learning models to predict the histological grading of DCIS. The findings highlight the significant role of calcification patterns, clinical factors, and the power of machine learning in enhancing diagnostic accuracy. Among the models tested, multinomial Naive Bayes showed numerically the best performance, although pairwise differences with XGBoost and logistic regression were not statistically significant, indicating comparable discriminative ability across the three models. Despite limitations such as the retrospective design, single-center cohort, absence of external validation, and reliance on radiologist-curated descriptors rather than raw images, this research underscores the promise of integrating machine learning into routine clinical workflows for more precise and personalized management of DCIS. The present findings should be interpreted as hypothesis-generating and require validation in external multi-center cohorts. Future work will focus on (i) external multi-center validation, (ii) integration of raw image-based deep learning and radiomics, and (iii) extension to clinically transformative endpoints such as invasive upgrade and recurrence prediction.

## Data Availability

The original contributions presented in the study are included in the article/**Supplementary Material**. Further inquiries can be directed to the corresponding author.
