# Supplementary material for: Prediction of histological grading in ductal carcinoma in situ based on mammographic signs and clinical information using machine learning models
Source: Front Oncol. 2026 Jul 2;16:1762400. doi: 10.3389/fonc.2026.1762400 (PMC13372783; doi:10.3389/fonc.2026.1762400)
Supplement: Supplementary file 1 [file Table1.docx]

**Supplementary Table S1. Inter-observer agreement for mammographic feature assessment.**

| **Feature category** | **Mammographic feature** | **No. of cases** | **Cohen's kappa** | **Agreement level** |
| --- | --- | --- | --- | --- |
| Calcification assessment | Presence of calcifications | 243 | 0.89 | Almost perfect |
| Calcification morphology | Round calcification | 243 | 0.84 | Almost perfect |
| Calcification morphology | Amorphous calcification | 243 | 0.76 | Substantial |
| Calcification morphology | Coarse heterogeneous calcification | 243 | 0.78 | Substantial |
| Calcification morphology | Fine pleomorphic calcification | 243 | 0.82 | Almost perfect |
| Calcification morphology | Linear or branching calcification | 243 | 0.86 | Almost perfect |
| Calcification distribution | Diffuse distribution | 243 | 0.81 | Almost perfect |
| Calcification distribution | Clustered distribution | 243 | 0.79 | Substantial |
| Calcification distribution | Regional distribution | 243 | 0.73 | Substantial |
| Calcification distribution | Segmental or linear distribution | 243 | 0.85 | Almost perfect |
| Associated mammographic finding | Mass lesion | 243 | 0.80 | Substantial |
| Associated mammographic finding | Architectural distortion | 243 | 0.71 | Substantial |

Note: Mammographic images were independently reviewed by two breast imaging radiologists who were blinded to histopathological results. Cohen's kappa was calculated for each BI-RADS-based mammographic descriptor before consensus adjudication. Kappa values of 0.61-0.80 indicate substantial agreement, and values of 0.81-1.00 indicate almost-perfect agreement.
