# Supplementary material for: Prediction of histological grading in ductal carcinoma in situ based on mammographic signs and clinical information using machine learning models
Source: Front Oncol. 2026 Jul 2;16:1762400. doi: 10.3389/fonc.2026.1762400 (PMC13372783; doi:10.3389/fonc.2026.1762400)
Supplement: Supplementary file 2 [file Table2.docx]

**Supplementary Table S2. Sensitivity analyses of feature selection methods.**

| **Feature/domain** | **Primary univariate filtering** | **LASSO regression** | **Recursive feature elimination** | **Final interpretation** |
| --- | --- | --- | --- | --- |
| Calcification morphology | Selected | Consistent | Consistent | Retained as a stable imaging feature domain. |
| Calcification distribution | Selected | Consistent | Consistent | Retained as a stable imaging feature domain. |
| Reproductive history | Selected | Consistent | Consistent | Retained as a clinical predictor. |
| Palpability of lesion | Selected | Consistent | Consistent | Retained as a clinical predictor. |
| Palpate hard texture | Selected | Consistent | Consistent | Retained as a clinical predictor. |
| Palpate edge clarity | Selected | Consistent | Consistent | Retained as a clinical predictor. |
| Palpation mobility | Not selected | Not consistently retained | Not consistently retained | Excluded from the final feature subset. |
| Age | Not selected | Not consistently retained | Not consistently retained | Excluded from the final feature subset. |
| Menstruation status | Not selected | Not consistently retained | Not consistently retained | Excluded from the final feature subset. |
| Lactation history | Not selected | Not consistently retained | Not consistently retained | Excluded from the final feature subset. |
| Family history | Not selected | Not consistently retained | Not consistently retained | Excluded from the final feature subset. |
| Nipple discharge | Not selected | Not consistently retained | Not consistently retained | Excluded from the final feature subset. |

Note: Selected features were identified using the primary univariate filtering strategy. LASSO regression and recursive feature elimination were used as sensitivity analyses to assess the robustness of the selected feature subset.
