# Supplementary material for: Prediction of histological grading in ductal carcinoma in situ based on mammographic signs and clinical information using machine learning models
Source: Front Oncol. 2026 Jul 2;16:1762400. doi: 10.3389/fonc.2026.1762400 (PMC13372783; doi:10.3389/fonc.2026.1762400)
Supplement: Supplementary file 3 [file Table3.docx]

**Supplementary Table S3. Preliminary model screening and final classifier selection.**

| **Model** | **Role** | **Algorithmic paradigm** | **Training set AUC (95% CI)** | **5-fold CV AUC (Mean +/- SD)** | **Test set AUC (95% CI)** | **Accuracy** | **Sensitivity** | **Specificity** | **Selection decision** |
| --- | --- | --- | --- | --- | --- | --- | --- | --- | --- |
| XGBoost | Final model | Tree-based ensemble | 0.788 (0.744, 0.832) | 0.776 +/- 0.041 | 0.763 (0.709, 0.818) | 0.761 | 0.726 | 0.725 | Retained for final comparison. |
| Logistic regression | Final model | Linear classifier | 0.796 (0.752, 0.840) | 0.781 +/- 0.038 | 0.756 (0.705, 0.807) | 0.758 | 0.824 | 0.692 | Retained as an interpretable baseline. |
| Multinomial Naive Bayes | Final model | Probabilistic classifier | 0.806 (0.761, 0.851) | 0.789 +/- 0.036 | 0.784 (0.735, 0.833) | 0.776 | 0.808 | 0.744 | Retained for final comparison. |
| Support Vector Machine | Preliminary model | Margin-based classifier | - | - | - | - | - | - | Not retained because it did not improve overall performance. |
| Random Forest | Preliminary model | Bagged tree ensemble | - | - | - | - | - | - | Not retained because it did not improve overall performance. |

Note: AUC, accuracy, sensitivity, and specificity were used to evaluate classifier performance. XGBoost, logistic regression, and multinomial Naive Bayes were retained for final comparative analysis.
