# Supplementary material for: Prediction of histological grading in ductal carcinoma in situ based on mammographic signs and clinical information using machine learning models
Source: Front Oncol. 2026 Jul 2;16:1762400. doi: 10.3389/fonc.2026.1762400 (PMC13372783; doi:10.3389/fonc.2026.1762400)
Supplement: Supplementary file 4 [file Table4.docx]

**Supplementary Table S4. Comparison of machine-learning models with radiologist-only baseline on the test set.**

| **Approach** | **Input information** | **Test set AUC (95% CI)** | **Accuracy** | **Sensitivity** | **Specificity** | **Accuracy difference vs baseline** | **Sensitivity difference vs baseline** | **Specificity difference vs baseline** |
| --- | --- | --- | --- | --- | --- | --- | --- | --- |
| Radiologist-only baseline | Mammographic features only | - | 0.673 | 0.704 | 0.636 | Reference | Reference | Reference |
| XGBoost | Mammographic and clinical variables | 0.763 (0.709, 0.818) | 0.761 | 0.726 | 0.725 | +0.088 | +0.022 | +0.089 |
| Logistic regression | Mammographic and clinical variables | 0.756 (0.705, 0.807) | 0.758 | 0.824 | 0.692 | +0.085 | +0.120 | +0.056 |
| Multinomial Naive Bayes | Mammographic and clinical variables | 0.784 (0.735, 0.833) | 0.776 | 0.808 | 0.744 | +0.103 | +0.104 | +0.108 |

Note: The radiologist-only baseline was based on mammographic interpretation without clinical variables or model output. Specificity for the radiologist-only baseline was calculated from the reported test-set composition.
